# The Effects of Alcohol Intoxication on Accuracy and the Confidence–Accuracy Relationship in Photographic Simultaneous Line‐ups

**DOI:** 10.1002/acp.3332

**Published:** 2017-06-27

**Authors:** Heather D. Flowe, Melissa F. Colloff, Nilda Karoğlu, Katarzyna Zelek, Hannah Ryder, Joyce E. Humphries, Melanie K.T. Takarangi

**Affiliations:** ^1^ School of Sport, Exercise and Health Sciences Loughborough University Loughborough UK; ^2^ College of Medicine, Biological Sciences and Psychology University of Leicester Leicester UK; ^3^ Department of Psychology Edge Hill University Ormskirk UK; ^4^ School of Psychology Flinders University Adelaide Australia

## Abstract

Acute alcohol intoxication during encoding can impair subsequent identification accuracy, but results across studies have been inconsistent, with studies often finding no effect. Little is also known about how alcohol intoxication affects the identification confidence–accuracy relationship. We randomly assigned women (*N* = 153) to consume alcohol (dosed to achieve a 0.08% blood alcohol content) or tonic water, controlling for alcohol expectancy. Women then participated in an interactive hypothetical sexual assault scenario and, 24 hours or 7 days later, attempted to identify the assailant from a perpetrator present or a perpetrator absent simultaneous line‐up and reported their decision confidence. Overall, levels of identification accuracy were similar across the alcohol and tonic water groups. However, women who had consumed tonic water as opposed to alcohol identified the assailant with higher confidence on average. Further, calibration analyses suggested that confidence is predictive of accuracy regardless of alcohol consumption. The theoretical and applied implications of our results are discussed.© 2017 The Authors *Applied Cognitive Psychology* Published by John Wiley & Sons Ltd.

Owing to the nature of their crimes, sexual assault assailants leave behind biological evidence more often than other assailants. DNA testing of this evidence can lead to apprehension and conviction of the guilty. It can also exonerate the innocent: To date, 349 men, convicted primarily of sex offenses, have had their convictions overturned (www.innocenceproject.org/dna‐exonerations‐in‐the‐united‐states). In the vast majority of these cases, at least one eyewitness or victim implicated the defendant, by positively identifying him from a line‐up. A recent review found that the majority of mistaken identifications involved witnesses or victims who expressed, at the time of the identification, low confidence in the likely accuracy of their identification (Garrett, 2011). These real world cases highlight the importance of studying the factors that influence identification accuracy and confidence, particularly in sex offenses. However, only two research studies to date have examined line‐up identification accuracy in this context. In these studies, participants watched a video that depicted a male sexually assaulting a woman (Yarmey, 1986; Yarmey & Jones, 1983). This work found that while the opportunity to view the assailant was associated with accuracy (Yarmey, 1986), the certainty of the identification did not relate to accuracy (Yarmey, 1986; Yarmey & Jones, 1983).

The present study extends this small literature, focusing on women's ability to identify a hypothetical sexual assault assailant from a line‐up. Further, an estimated 35–72% of victims in sexual assault cases are alcohol‐intoxicated during the offense (Mohler‐Kuo, Dowdall, Koss, & Wechsler, 2004; Office for National Statistics, 2015). Therefore, to increase the relevance of our work to line‐up identifications in rape cases, we varied whether women were under the influence of alcohol during encoding, assessing whether alcohol intoxication decreases accuracy. We also examined the confidence–accuracy relationship as a function of alcohol intoxication. If confidence is predictive of accuracy, confidence should be relatively low when the witness identifies the wrong person, and it should be relatively high when the witness correctly identifies the perpetrator. In the sections that follow, we review the relevant research background and outline our predictions.

## Background: The Effects of Alcohol on Identification Accuracy

Police surveys and archival research indicate that intoxicated witnesses and victims often provide police statements and take identification tests in criminal investigations (Evans, Schreiber Compo, & Russano, 2009; Palmer, Flowe, Takarangi, & Humphries, 2013). Yet, relatively few line‐up studies have examined the impact of alcohol on identification accuracy (Hagsand, Roos af Hjelmsäter, Granhag, Fahlke, & Söderpalm‐Gordh, 2013; Harvey, Kneller, & Campbell, 2013; Kneller & Harvey, 2016; Yuille & Tollestrup, 1990). This handful of studies—summarized in Table 1—show that alcohol intoxication during encoding has no effect on accuracy in simultaneous line‐ups (Hagsand et al., 2013; Kneller & Harvey, 2016; Yuille & Tollestrup, 1990). Dysart, Lindsay, MacDonald, and Wicke (2002), however, found that identification accuracy was lower in perpetrator absent, but not perpetrator present, showups. In a showup 1Participants in this study were also given a simultaneous line‐up at a later date via email, but interpretation of these results is limited because of the relatively low response rate., the suspect is presented to the witness alone, without any fillers. Participants in the study by Dysart et al. learned and were tested on their memory for a person while they were either alcohol‐intoxicated or sober. The authors argued that these results are in line with alcohol myopia theory, which proposes that, when people are under the influence of alcohol, attention is allocated to the most immediate and salient cues in the environment, whereas peripheral and weaker cues that conflict with salient cues receive less attention (Steele & Josephs, 1990). They reasoned that intoxicated participants in their study focused on a salient feature (i.e., hairstyle) of the ‘culprit’, which led them to misidentify the innocent suspect in the perpetrator absent showup who had a similar feature. However, a recent study found that participants who were intoxicated during encoding were no more likely than sober participants to be influenced by distinctive features during a basic face recognition task, regardless of whether they were intoxicated versus sober at test (Colloff & Flowe, 2016). In this study, participants who had studied faces while intoxicated were more likely to false alarm to any face, with or without a distinctive feature, suggesting that intoxicated participants were basing their recognition decisions on familiarity rather than identity‐based information.

**Table 1:** Proportion of accurate identifications as a function of alcohol condition reported in the line‐up literature

	No alcohol control group				Alcohol group				
Study	PP accuracy	PP *n*	PA accuracy	PA *n*	PP accuracy	PP *n*	PA accuracy	PA *n*	Mean BAC
Hagsand et al. (2013)t	0.25	20	0.24	21	0.23	40	0.40	42	0.04–0.08%
Kneller and Harvey (2016)c	0.27	40	0.45	40	0.35	20	0.45	20	0.05%
Harvey et al. (2013)	0.37	30	0.70	30	0.3	30	0.70	30	0.11%
Yuille and Tollestrup (1990)	0.89	35	0.76	33	0.91	22	0.61	23	0.10%

*Note*: PP, perpetrator present; PA, perpetrator absent; t, there were two alcohol groups, we collapsed across them here for simplicity; c, there were two control groups, we collapsed across them here for simplicity.

Given this mixed picture, we conducted the present study to examine whether alcohol intoxication during encoding decreases line‐up identification accuracy. We extended previous work by presenting the perpetrator within a hypothetical sexual assault scenario and by employing a balanced placebo design that controlled for alcohol expectancy. According to alcohol expectancy theory, people may try to compensate for alcohol's negative effects on cognition by increasing their attention, and this increased attention may mediate the effect of alcohol on cognition (Testa et al., 2006). With respect to memory performance, alcohol expectancy does not seem to have an effect (Hull & Bond, 1986). However, there is some evidence that alcohol expectancy in situations that are associated with an increased risk of sexual assault may lead women to be hypervigilant and respond with increased caution (see Testa et al., 2006), which could allow them to compensate for any negative influence of alcohol on memory for the perpetrator. To investigate this issue in the context of line‐ups, in the present study, we controlled for alcohol expectancy and assessed its effect on accuracy and the confidence–accuracy relationship. We used calibration techniques to do so, which have not been employed before. We review this literature next.

## Alcohol and Metamemory Judgments of Line‐up Identification Accuracy

According to models such as trace access theory (Burke, Mackay, Worthley, & Wade, 1991; also see Green & Swets, 1966; Macmillan & Creelman, 1991), people monitor their memory processes. When making recognition judgments, they evaluate the contents of recognition memory and give higher confidence ratings to items that have greater memory strength. Thus, if memory strength underpins both confidence and accuracy, then they should be positively correlated. A review of early research indicated that the correspondence between confidence and accuracy was weak (Wells & Murray, 1984). However, subsequent research has found that confidence predicts accuracy when there is sufficient variability across witnessing conditions to allow for detecting the association (Lindsay, Read, & Sharma, 1998). Sporer, Penrod, Read, and Cutler (1995) found in their meta‐analyses of the line‐up identification literature that the confidence–accuracy correlation is positive, particularly for people who identify a face from the line‐up (i.e., ‘choosers’). They noted that the magnitude of the relationship between confidence and accuracy was stronger than many other predictors of accuracy studied by eyewitness researchers, including weapon focus and own race bias. Further, a number of researchers have argued recently, on the basis of empirical data, that confidence is predictive of line‐up identification accuracy (e.g., Brewer & Wells, 2006; Palmer, Brewer, Weber, & Nagesh, 2013; Sauer, Brewer, Zweck, & Weber, 2010; Wixted & Wells, in press; Wixted, Mickes, Clark, Gronlund, & Roediger, 2015; Roediger, Wixted, & DeSoto, 2012). In a recent reappraisal of confidence–accuracy literature, Wixted and Wells (in press) conclude that confidence is strongly associated with identification accuracy in adults, provided it is taken under pristine conditions (e.g., the composition of the line‐up is fair). Thus, there is now a sizable body of work and a consensus that confidence can be predictive of line‐up identification accuracy.

There are factors that can affect the confidence–accuracy relationship, however. According to the optimality hypothesis, confidence is predictive of accuracy when information‐processing conditions are optimal during memory encoding (e.g., relatively long duration of exposure, better viewing opportunity), storage, and retrieval (Bothwell, Deffenbacher, & Brigham, 1987; Deffenbacher, 1980). As another example, under some conditions in which remembering is more difficult (e.g., relatively short duration of exposure, long retention interval between learning and test), people can be overconfident in the accuracy of their line‐up identification (Palmer, Brewer et al., 2013). The confidence–accuracy relationship also can be weakened when people fail to take into account factors that might have negatively affected their accuracy (see Hourihan, Benjamin, & Liu, 2012 and Mickes, 2015) and when people receive feedback from the line‐up administrator regarding their identification accuracy (e.g., Wells & Bradfield, 1998). Therefore, in evaluating witness confidence to predict likely accuracy, the totality of the circumstances (i.e., from the time of the crime, to the time of the in court identification) needs to be taken into account.

How might alcohol affect meta‐memory judgments and, specifically, confidence–accuracy calibration? There is limited evidence on this issue. When answering general knowledge questions, participant confidence does not appear to be affected by alcohol intoxication (Nelson, McSpadden, Fromme, & Marlatt, 1986). In the context of line‐ups, Yuille and Tollestrup (1990) found intoxicated compared with sober participants reported lower levels of confidence for incorrect compared with correct identifications. Further, they found that confidence and accuracy were significantly associated and *more* strongly related for intoxicated compared with sober participants, suggesting meta‐memory judgments are superior if people are under the influence of alcohol compared with sober during encoding. These results run counter to the optimality hypothesis, which would predict a stronger relationship between confidence and accuracy for sober compared with intoxicated participants. So why might alcohol intoxication during encoding paradoxically strengthen the confidence–accuracy relationship? One possibility is that people are aware that alcohol intoxication can compromise their memory, and this awareness attenuates overconfidence. Palmer, Brewer et al. (2013) proposed and found evidence suggesting that participants can make ‘theory‐based’ confidence judgments. If it is apparent to participants that a given factor weakens their memory (e.g., divided attention during encoding), they will still be calibrated because they take this theory‐based information into account when evaluating their confidence. Applied here, we might predict that alcohol intoxication during encoding reduces overconfidence and thereby strengthens the confidence–accuracy relationship for those who are intoxicated versus sober during encoding. To elaborate, people who are intoxicated during encoding might be better calibrated because they take into account theory‐based information regarding the negative effects of alcohol on memory. This explanation would account for why Yuille and Tollestrup (1990) found that confidence was more predictive of accuracy for intoxicated compared with sober participants.

Yuille and Tollestrup's (1990) results have not been replicated, however; other identification studies assessing the confidence–accuracy relationship report null findings (Dysart et al., 2002; Hagsand et al., 2013; Harvey et al., 2013; Kneller & Harvey, 2016). Evidence on the issue is also limited by previous studies having employed the point‐biserial correlation coefficient (r_pb_) to examine the effects of alcohol on the confidence–accuracy relationship. Juslin, Olsson, and Winman (1996) demonstrated that the confidence–accuracy relationship is poorly estimated by r_pb_ and proposed that researchers use a calibration approach instead. Calibration entails associating objective probabilities of accuracy with subjective estimates of accuracy, such as confidence. There are a number of calibration approaches (i.e., calibration curves, over/under confidence statistics, c statistic, Normalized Resolution Index). For example, calibration curves plot the proportion of accurate identifications at each level of confidence. Participants are well‐calibrated to the extent that accuracy and confidence correspond across the range of the confidence scale. For example, calibration is perfect if there is a 1:1 correspondence between confidence and accuracy, with participants who report 10% confidence being 10% accurate, those who report 30% confidence being 30% accurate, and those who report 50% confidence being 50% accurate, and so on. Calibration research finds that confidence can be predictive of identification accuracy, even when memory is relatively weak (e.g., Sauer et al., 2010). Even when calibration is perfect, r_pb_ can severely underestimate the strength of the relationship between confidence and accuracy because its size is affected by the underlying variability of the confidence distribution (Juslin et al., 1996). Therefore, here we extend the alcohol literature by employing a calibration approach to compare the confidence–accuracy relationship in participants who were intoxicated versus sober during encoding.

We predicted lower identification accuracy and lower confidence for participants who had consumed alcohol as opposed to tonic water. We also tested whether alcohol consumption and/or alcohol expectancy (i) weakens the confidence–accuracy relationship, as per the optimality hypothesis, or (ii) strengthens the confidence–accuracy relationship as per Palmer, Brewer et al.'s (2013) theory‐based information model of the confidence–accuracy relationship.

## Method

### Participants

We recruited 153 female participants, aged 18–32 years (*M* = 20.53, *SD* = 2.28) from the University of Leicester. The research protocol was reviewed and approved by the University of Leicester's Psychology Research Ethics Committee. Written informed consent was obtained from participants directly, prior to their participation. Our participants were all young adult women, because sexual assault disproportionately affects this group (U.S. Department of Justice, Office of Justice Programs, Bureau of Justice Statistics, 2010).

### Design

We employed a 2 beverage (consumed tonic water or alcohol) × 2 expectancy (told consumed tonic water or alcohol) × 2 perpetrator (perpetrator present or perpetrator absent) between participants design. Women were randomly assigned to conditions. The outcome measures were identification response (identify the perpetrator, identify a filler, or not identify anyone, i.e., reject line‐up), choosing (choosers or nonchoosers, depending on whether a line‐up member was identified as the perpetrator from the line‐up), identification accuracy (correct or incorrect line‐up response), and confidence.

The study was conducted over 2 years. In the first year, we used a 1‐day retention interval between encoding and the line‐up test (*n* = 80), and in the second year, we used a 7‐day retention interval (*n* = 73). Consequently, because women were not randomly assigned to a retention interval condition, if we found any effects of retention interval on accuracy, we would have to interpret this with caution.

During the 2‐year study duration, we recruited as many participants as possible. We randomly assigned 75 women to the tonic water condition (38 were randomly assigned to the perpetrator present condition and 37 to the perpetrator absent condition) and 80 to the alcohol condition (42 were randomly assigned to the perpetrator present condition and 38 to the perpetrator absent condition). Assuming a medium effect size for beverage, 80% power, and an alpha level of .05, we needed 31 participants in the tonic water condition and 31 participants in the alcohol condition to detect a significant effect of beverage on accuracy within the perpetrator present and/or perpetrator absent condition. Our cell sizes exceeded these numbers. 2The only line‐up study to have found an effect of alcohol on identification accuracy is Read, Yuille, and Tollestrup (1992). Their participants acted in the role of perpetrator rather than witness. Nevertheless, the effect size for beverage in target absent line‐ups under low arousal was medium in size (Cramer's *V* = .26, *df* = 1), and we used this number to guide us in terms of estimating effect size. Further, our sample size allowed for detecting a medium sized effect of expectancy on accuracy within the alcohol beverage condition (i.e., within the alcohol condition, 40 participants expected alcohol and 38 participants expected tonic water) and within the tonic water beverage condition (i.e., within the tonic water condition, 40 participants expected alcohol, and 35 expected tonic water). Our sample size was also adequate (assuming 80% power, .05 alpha) for detecting a medium size effect of beverage or expectancy on confidence ratings based on data reported by Yuille and Tollestrup (1990). Given these parameters, a total of 52 participants were required in each cell to detect a significant difference between participants who had and had not consumed alcohol, and participants who expected versus did not expect to consume alcohol. Note that we did not make any predictions about perpetrator presence in the line‐up or whether it would differentially affect accuracy or confidence depending on the combined effect of beverage with expectancy; hence, we did not include the perpetrator condition and the interaction between beverage and expectancy in power calculations.

### Materials and procedure

We circulated around the University campus an advertisement for female social drinkers. The advertisement indicated that participants may consume alcohol as part of the study and that the topic concerned sexual and dating behaviors. Women who contacted the researchers received further information via an email, which explained that participants had to successfully complete a pre‐screening, reiterated that the study was about sexual and dating behaviors, and stressed that participation may also entail discussions about sexual assault. We specified this information so that women could elect to participate with knowledge about the topic of the study. We invited interested participants to complete the pre‐screening, which was accessible via a link to an online survey that we provided in the email.

The pre‐screening included the Alcohol Use Disorders Identification Test (AUDIT), which was developed by the World Health Organization to assess whether a person's drinking is harmful, hazardous, or dependent (Babor, Higgins‐Biddle, Saunders, & Monteiro, 2001; Saunders, Aasland, Babor, de la Fuente, & Grant, 1993) and a general health questionnaire, which we devised, that asked women to indicate any prescription drugs they were currently taking and any health problems they were experiencing. We invited women to participate if they scored less than 11 on the AUDIT and if they also indicated that they did not have any health problems and did not use any prescription drugs that would cause an adverse reaction to alcohol. Among the participants who met these criteria, their mean AUDIT score was 6.00 (*SD* = 2.55). We asked participants not to consume any alcohol on the day of the study, nor any food during the 4 hours prior to their participation time; to the best of our knowledge, participants followed this instruction.

Women participated individually. When the participant reported to the laboratory, the experimenter confirmed her answers on the AUDIT and the general health questionnaire. The participant then took a urine‐based pregnancy test to confirm that she was not pregnant. The experimenter then told the participant that she could not be released from the laboratory that day until her blood alcohol content (BAC) level was less than 0.02%. If she elected to leave before then, the researchers would call her a taxi to transport her home. She was also advised to not drive an automobile or to not operate heavy machinery for the rest of the day. She then signed a form to indicate that she understood the conditions under which she would be released.

We used a portable breathalyzer (manufactured by AlcoHAWK) to confirm that the participant's BAC was 0.00%. Next, the experimenter provided the participant with three cups, which either contained an alcoholic or a tonic water beverage, depending on the beverage and expectancy conditions to which she had been assigned. In the alcoholic beverage conditions, we dosed women with five parts tonic water to one part vodka to achieve a BAC of 0.08%, based on their height and weight (i.e., hence, the amount of alcohol consumed varied across participants depending on their height and weight), following the procedures of previous research (e.g., Curtin & Fairchild, 2003). This calculation translates into an average alcohol dose of 35.67 g (*SD* = 3.63 g) across participants in the alcohol group. It should also be noted that laboratory research investigating the effects of alcohol on cognition and behavior does not typically employ dosage levels that result in a BAC over 0.08%. Ethics Committees usually do not permit higher dosage levels for insurance reasons and to reduce the possibility of serious adverse events occurring in the laboratory. In the tonic water beverage conditions, we gave women three cups of tonic water. In both the alcohol and tonic water conditions, the cups contained vodka soaked limes and were rimmed with vodka; here, our aim was to disguise the alcohol condition to which women had been assigned. As per instruction, all participants consumed each cup within 5 minutes.

To control for any psychological expectancy effects of alcohol and separate them from the pharmacological effects of alcohol, we used the balanced‐placebo design in which half of the participants in each beverage condition were told that they were going to consume alcohol, whereas the other half were told that they were going to consume tonic water only. Additionally, the cups participants received were labeled with ‘tonic’ or ‘vodka and tonic’, depending on the expectancy condition to which they had been assigned.

Thirty minutes later, we administered the scenario. At this time, mean BAC in the tonic water group was 0.00% (*SD* = 0.00) and 0.08% (*SD* = 0.01) in the alcohol group. We measured the participant's BAC level at several time points while she was in the laboratory. Time points were approximately every 15 minutes, but as we were measuring participants between tasks, we occasionally had to wait for participants to finish a given task before taking the reading. On average, BAC was at its peak level by the time women engaged in the scenario. All women began the scenario after 30 minutes, even if they had not reached 0.08%. The participant assigned herself a five‐digit personal identification number (PIN), which she used when starting the scenario. The PIN was also recorded on the alcohol consumption worksheet. The purpose of the PIN was to link participant behavior in the scenario to the line‐up identification data.

The scenario was written for the purpose of this study, following the *Participant Choice* procedure (Flowe, Ebbesen, & Putcha‐Bhagavatula, 2007; Flowe, Stewart, Sleath, & Palmer, 2011). This procedure heightens the participant's personal involvement in the scenario. It allows the participant to determine for herself whether or not she interacts with the man depicted and whether or not she consents to any sexual activity with him. We wrote multiple versions of the scenario to increase the generality of the findings. We randomly assigned participants to read one of 16 possible versions, which we composed by crossing four locations (i.e., bar, her house, his house, and a party) with four different versions of the man (i.e., each version had unique biographical information about the man, such as his occupation, the type of car he drove, his hometown, and his hobbies). The basic plot of the scenario was the same across the versions: The participant encounters a man at a location, and soon he begins making romantic overtures towards her.

The scenario was computer‐based, presented in several stages depending on the participant's choices. At the start of the program, the participant entered her PIN. Then, during the first stage, the scenario opens with an introduction to the setting and a description of the participant meeting a man for the first time; a photo of him (i.e., a head and shoulder shot) accompanies the text. The male portrayed was one of four possible men (all were Caucasian and aged 25 years on average), with participant randomly assigned to see one of them. The photograph of the man was visible during only the first stage. The man introduces himself and describes his music interests, occupation, and hobbies. He behaves flirtatiously towards the participant. The participant was then given her first choice: to continue to interact with the man (e.g., accept a ride home from him) or to ‘call it a night’.

If the participant elected to remain in the scenario, the next stage of the scenario is presented (e.g., ‘He begins to rub your back’.). Following this text, the participant was asked whether she wants to remain in the situation being described or if she wants to ‘call it a night.’ If she remains, another stage of the scenario is presented. If the participant remained in the scenario long enough, she would read that she and the man have arrived, alone, at either her house or the man's house (depending on scenario version). At this location, sexual activity was depicted. For as long as the participant chose to remain in the scenario, the sexual activities were described as consensual. If she remained in the scenario until the end, the final activity described was consensual sexual intercourse. The participant could not move backwards through the scenario; once she made a decision, she could not change it, nor could she return to a previous part of the scenario.

If the participant elected at any point to ‘call it a night’, the rape scenario was presented. If she withdrew before they were alone at her house or the man's house, the participant was told that she and the man parted company after she ‘called it a night’, but later, the man broke into her house, restrained her, and committed a legally definable act of rape against her. If she chose to ‘call it a night’ after they were alone at her house or the man's house, the participant was told he had refused to take ‘no’ for an answer, restrained her, and committed a legally definable act of rape against her. We provided relatively little descriptive detail about the rape itself because, for ethical reasons, we did not want to subject the participant to gratuitous violence. Rather, our sole aim in wording the continuation was to make it as clear as possible that a legally definable act of rape had taken place.

After the participant engaged in the scenario, she indicated whether she thought the event was rape and whether she would report it to the police as rape, using a Likert‐type scale, anchored at, 1, *Definitely No*, and 11, *Definitely Yes.* We required every participant to remain in the laboratory for at least 2 hours to make it more difficult for a placebo participant to correctly guess her beverage condition. Participants who consumed alcohol were released after their BAC was less than 0.02%.

Twenty‐four hours or 7 days later, the participant was emailed with a link to an online survey that presented a six‐person simultaneous line‐up test. It is important to note that we often tested women towards the end of the week (Thursday or Friday) owing to participants' availability. Our lab is not open on weekends; hence, in order to test women 24 hours or 7 days later, we had to administer the line‐up test online. Line‐up identification studies have reported similar findings for participants tested in the laboratory compared with online (Flowe, Smith, Karoğlu, Onwuegbusi, & Rai, 2016; Gronlund, Carlson, Dailey, & Goodsell, 2009; Mickes, Flowe, & Wixted, 2012). Additionally, studies investigating other cognitive phenomena (e.g., stroop, attentional blink) have reported similar results across laboratory versus online administrations (e.g., Crump, McDonnell, & Gureckis, 2013).

The line‐up was presented in two rows of three faces. In the perpetrator present condition, the male from the scenario was in either position two or position five and surrounded by five fillers who matched his description. The photographs were selected from the Radboud face database (Langner et al., 2010). The individuals in the line‐up did not have distinguishing features, were all wearing black t‐shirts, were the same age, race and build, and had the same hair color. A number appeared beneath each photo. The photograph of the male shown during the scenario was different from the photograph of him that was presented in the perpetrator‐present line‐up. In the perpetrator‐absent version of the line‐up, the male from the scenario was not present; he was replaced with a filler. To test that our line‐ups were fair and the perpetrator did not stand out from the fillers, we used the mock witness procedure. To do this, we recruited another group of participants (*N* = 37), gave them only a written description of the perpetrator (i.e., hair color, eye color, build, height, and facial hair), and asked them to identify the person who they thought was the police suspect. We calculated E′, which measures effective size, or the number of line‐up members who adequately resemble the suspect (Tredoux, 1998). Across the line‐ups, E′ ranged from 4.24 to 5.35 (*M* = 4.78, *SD* = 0.43), which is comparable with the nominal size of the line‐ups (i.e., six persons or the actual number of line‐up members), as well as effective sizes reported in previous research in the lab (Gronlund et al., 2009). These data suggest that our line‐ups were fair.

We warned participants, as per line‐up administration guidelines (Technical Working Group for Eyewitness Evidence, 1999), that the man from the scenario might not be present in the line‐up. We cautioned them that not selecting anyone could be the correct answer. The purpose of cautionary instructions like these is to minimize positive identifications based on guessing. The participant indicated her identification response by either identifying a person by number, indicating that the male from the scenario was ‘Not there’, or by indicating ‘I don't know’. Identification accuracy is higher when the ‘I don't know’ option is given compared with not given (Weber & Perfect, 2012). In analyzing the data, ‘I don't know’ and ‘Not there’ responses were treated as line‐up rejections. Following the line‐up test, we asked participants to indicate how confident they were in the accuracy of their response using a 0–100% confidence scale. After submitting their identification responses, participants were asked what drink they thought they had consumed, and to indicate how intoxicated they felt when reading the scenario using a Likert‐type scale, anchored from 0, *Completely Sober*, to 10, *Completely Intoxicated.* We asked women to report their beliefs at the conclusion of the study because we wanted to maintain alcohol expectancies in line with what we had told them during the course of the study.

Finally, we arranged for the participant to return to the lab for debriefing—more often than not, she came in more than a week after her participation, owing to tight schedules, and she was remunerated £4 for every hour that she participated.

## Results

### Preliminary analyses

We assessed whether beverage and expectancy affected the stage at which women withdrew from the scenario. A 2 beverage × 2 expectancy analysis of variance (ANOVA) with stage as the dependent variable indicated no significant effects. Women tended to remain in the scenario longer if they had been given alcohol (*M* = 12.16, *SEM* = .87 stages) compared with tonic water (*M* = 10.07, *SEM* = .89 stages), but the difference was not statistically significant, *F*(1, 149) = 2.81, *p* = .10. Further, women who expected alcohol tended to remain in the scenario as long as women who expected tonic water only (*M* = 11.65, *SEM* = .86 stages versus *M* = 10.58, *SD* = .90 stages, respectively), *F*(1, 149) = .73, *p* = .39, and the expectancy × beverage interaction was not significant, *F*(1, 149) = 1.11, *p* = .29. Participants tended to believe that the sexual intercourse that took place was not consensual (1–11 rating scale, with 1 indicating it was definitely not consensual, and 11 indicating it was definitely consensual: *M* = 2.50, *SD* = 3.26) and to think that they would report the culprit's details to the police if the scenario had happened to them in real life (1–11 rating scale, with 1 indicating she definitely would not report, and 11 indicating she definitely would report: *M* = 8.77, *SD* = 3.38). Overall, 86% (*n* = 133) of participants read the scenario continuation (i.e., they ‘called it a night’ at some point in the scenario, and therefore, they read the rape depiction). On average, participants typically ‘called it a night’ at stage 11 (*M* = 11.20, *SD* = 7.73 stages), which meant that participants tended to consent to kissing and a back rub but no other sexual activity. Line‐up identification results did not vary in relation to whether women read the scenario continuation or in relation to the average stage at which they withdrew from the scenario; hence, to maximize our statistical power, the results we report hereafter are based on the entire sample (*n* = 153).

For the first 56 participants (*n* = 28 alcohol, *n* = 28 tonic water), we recorded the length of time women spent on the first scenario stage, which was when the photo of the male actor was displayed. We inadvertently did not record this information for the other participants. There was no difference across the beverage groups in the average length of time that women spent on the first stage (alcohol *M* = 10.62 s, *SD* = 7.42 s versus tonic water *M* = 8.89 s, *SD* = 5.15 s), *t*(54) = 1.10, *p* = .32.

Next, we turned to our subjective measures of alcohol intoxication. First, we examined whether women felt more intoxicated depending on beverage and expectancy condition. A 2 (beverage) × 2 (expectancy) between subjects ANOVA revealed that women who consumed alcohol reported feeling more intoxicated compared with those who consumed tonic water (*M* = 5.14, *SEM* = .29 versus *M* = 1.76, *SD* = .30, respectively, a significant main effect for beverage, *F*(1, 149) = 64.32, *p* < .0001, η_p_^2^ = .30. Women who expected alcohol reported feeling more intoxicated compared with those who expected tonic water (*M* = 3.63, *SEM* = .29 versus *M* = 3.27, *SEM* = .30, respectively), but the difference was not statistically significant, *F*(1, 149) = 0.78, *p* = .38. Beverage and expectancy did not interact, *F*(1, 149) = 0.70, *p* = .40.

Next, we examined women's responses regarding the beverage that they thought they had consumed. Across the whole sample, 63% thought they had consumed alcohol, indicating that participants tended to think they had been given alcohol. The expectancy manipulation was significantly associated with women's responses regarding what beverage they thought they had consumed, *χ* ^2^ = 14.37, *p* < .001, Φ = .30. We also analyzed whether the beverage women had actually consumed affected their thoughts regarding the beverage they believed they had consumed. Women's responses regarding the beverage they thought they had consumed were significantly associated with beverage condition, *χ* ^2^ = 27.25, *p* < .001, Φ = .42, with 83% of women in the alcohol condition reporting they thought they had consumed alcohol, and 57% in the tonic water beverage condition reporting they thought they had consumed tonic water. Thus, women were more likely to think they had consumed alcohol if they actually did consume alcohol.

We also assessed the relationship between the beverage women thought they had consumed in relation to the alcohol expectancy manipulation. In the alcohol beverage condition, there was no association between the beverage that women thought they had consumed and expectancy: 90% of those who had been told alcohol said they thought they had been given alcohol and 76% of those who had been told tonic water said that they thought they had been given alcohol. These results indicate that women in the alcohol condition generally felt they had been given alcohol, regardless of what they were told. In the tonic water beverage condition, expectancy was significantly associated with the beverage that women thought they had consumed, *χ* ^2^ (1) = 17.47, *p* < .001, Φ = .48, with 65% of those who had been told alcohol saying that they thought they had been given alcohol and 82% of those who had been told tonic water saying that they thought they had been given tonic water. Taken together, the self‐report data suggest that the expectancy manipulation worked but only in the tonic water beverage condition. Therefore, in the analyses that follow, we included whether women thought they had consumed alcohol as another measure of expectancy.

### Alcohol and identification responses

Ideally, we would submit our identification data to a multilevel loglinear analysis, with beverage, expectancy, perpetrator, delay, and identification outcome as the factors. One of the assumptions of multilevel loglinear analysis is that the expected frequency is greater than 5 in 80% of the cells (Tabachnick & Fidell, 2013). Our data violated this assumption, in large part, because participants had a strong tendency to reject the line‐up when the perpetrator was absent and rarely chose a foil when the perpetrator was present (Table 2). Therefore, we instead conducted two logistic regression analyses, with choosing and accuracy as the outcome measures. We assessed the effects of beverage, expectancy, delay, and perpetrator in these analyses.

**Table 2:** Proportions of identification responses by beverage group, expectancy, and identification outcome

Expected alcohol			Expected tonic water		
Consumed tonic water			Consumed tonic water		
	PP	PA		PP	PA
	*(n = 23)*	*(n = 17)*		*(n = 15)*	*(n = 20)*
Perpetrator	0.61	—	Perpetrator	0.53	—
Filler	0.09	0.41	Filler	0.07	0.20
Reject	0.30	0.59	Reject	0.40	0.80

Expected alcohol			Expected tonic water		
Consumed alcohol			Consumed alcohol		
	PP	PA		PP	PA
	*(n = 19)*	*(n = 21)*		*(n = 23)*	*(n = 17)*
Perpetrator	0.42	—	Perpetrator	0.63	—
Filler	0.16	0.19	Filler	0.17	0.31
Reject	0.42	0.81	Reject	0.32	0.69

*Note.* PP, perpetrator present; PA, perpetrator absent.

#### Choosing

Results indicated that inclusion of the study factors produced a better fit compared with a model without any predictors, *χ* ^2^ (4) = 24.89, *p* < .001, Nagelkerke *R* ^2^ = .20. However, perpetrator was the only significant predictor in the model, β = −1.60, *SE* = .35, Wald (1) = 20.28, *p* < .001, Exp(*B*) = .20. Women were more likely to choose when the assailant was present rather than absent from the line‐up (65% versus 20%).

The beverage women thought they had consumed was not associated with choosing, *r* = .03, *p* = .74, *N* = 153.

#### Accuracy

The model did not fit better than one without any predictors, *χ* ^2^ (3) = 7.05, *p* = .13, Nagelkerke *R* ^2^ = .06. The only significant factor was perpetrator, Wald (1) = 4.69, *p* = .03, β = .76, *SE* = .35, Exp(*B*) = 2.13. Women were more accurate when the perpetrator was absent compared with present in the line‐up (73% versus 46%).

The beverage women thought they had consumed was not associated with accuracy, *r* = .02, *p* = .76, *N* = 153.

### The confidence–accuracy relationship

#### Mean confidence

Next, we examined confidence in relation to beverage and accuracy. Our first analysis examined whether confidence, on average, varied in relation to alcohol group and accuracy. We entered confidence into a 2 (beverage) × 2 (perpetrator) × 2 (expectancy) between subjects ANOVA. We included perpetrator in the analysis because it was a significant predictor of accuracy in the analyses reported earlier. Women in the alcohol group reported lower confidence than their tonic water counterparts (*M* = 58.08, *SEM* = 3.44, versus *M* = 68.21, *SEM* = 3.52, respectively), a significant main effect for beverage, *F*(1, 145) = 4.22, *p* = .04, η_p_^2^ = .03. No other main effects and no interaction effects were significant. Expectancy did not affect confidence levels (M_told alcohol_ = 62.37, *SD* = 31.35, versus M_told tonic water_ = 64.38, *SD* = 29.58), *t*(151) = .40, *p* = .68. We also analyzed whether the beverage women thought they had consumed affected confidence ratings and found no association, *F*(1, 151) = 0.54, *p* > .05.

In sum, alcohol consumption, but not alcohol expectancy, was associated with lower confidence levels overall. But, did confidence–accuracy calibration vary depending on whether participants had consumed alcohol? To address this question, we turned to our calibration tests.

#### Calibration analyses

**Figure 1:**
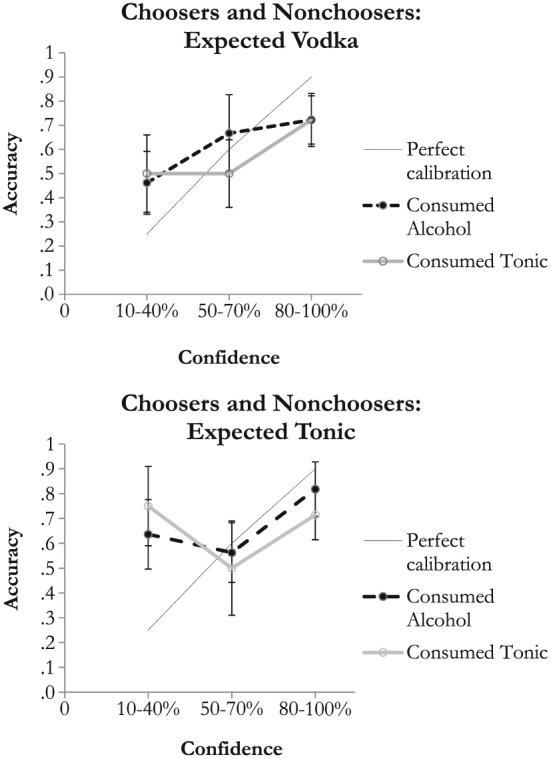
Mean accuracy by confidence level and beverage condition. Participants who were told they would consume vodka are plotted in the top panel, while those who were told tonic water are plotted in the bottom panel. The dashed line indicates perfect calibration. Error bars ±1 SEM

Figure 1 plots calibration curves for the alcohol and tonic water groups for choosers and nonchoosers, holding constant expectancy. The top panel shows the results for those who expected vodka, and the bottom panel shows the results for those who expected tonic water. We collapsed across confidence levels (the *x*‐axis) in the analysis to stabilize the functions. Each data point reflects the average accuracy rate that was observed for the given confidence level range. To assist with assessing overconfidence versus underconfidence, an ideal calibration line has been drawn. The point that is leftmost on the ideal line corresponds to the 10–40% confidence range. Here, 25% is plotted for accuracy. If calibration is perfect, average accuracy should be 25%, because 25% is the midpoint of the 10–40% range. Likewise, average accuracy ideally should be 60% for the 50–70% confidence range and 90% for the 80–100% confidence range. Note that when a data point falls above the ideal line, underconfidence is indicated (i.e., identification accuracy is higher on average than witnesses thought it would be given their confidence ratings), whereas when a data point falls below the perfect calibration line, overconfidence is indicated (i.e., identification accuracy is lower on average than witnesses thought it would be given their confidence ratings).

As can be seen in Figure 1, the pattern of results for those who consumed alcohol versus tonic is similar, both when participants expected vodka (top panel) and when they expected tonic (bottom panel). In all groups, there was a tendency towards underconfidence at the lowest confidence levels and overconfidence at the highest confidence levels, which is similar to past research. There is no evidence that alcohol consumption or expecting to consume alcohol resulted in worse calibration, however. Descriptively speaking, those who expected alcohol and consumed it were underconfident at the 50–70% level, while all other groups were overconfident. However, the overlapping error bars indicate that these differences are not statistically significant. For those who expected tonic (bottom panel), the confidence–accuracy relationship was also similar for those who consumed alcohol compared with those who did not. Further, the data plots in the top and bottom panels of Figure 1 were compared to assess possible expectancy effects. Again, the error bars overlapped, which indicates that expectancy did not systematically affect the confidence–accuracy relationship.

**Figure 2:**
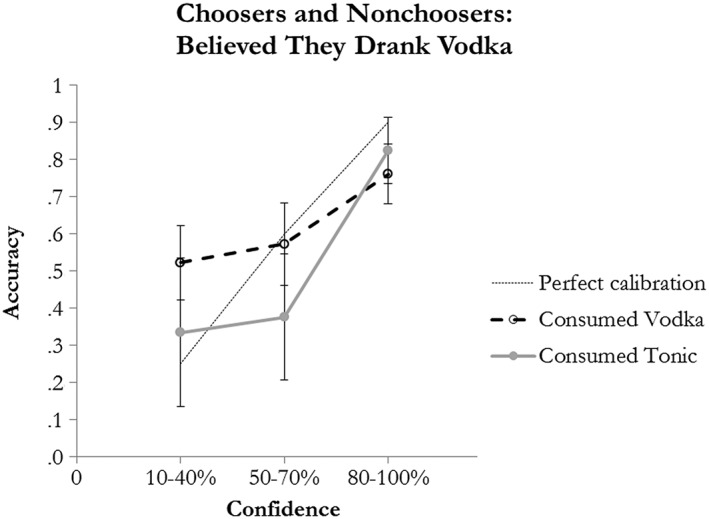
Mean accuracy by confidence level and beverage condition for participants who believed that they would consume vodka. The dashed line indicates perfect calibration. Error bars ±1 SEM

We also examined the confidence–accuracy relationship as a function of beverage separately for those participants who indicated that they thought that they had consumed alcohol, and these data are reported in Figure 2. As shown, the error bars overlap, once again demonstrating that the confidence–accuracy relationship was similar across beverage groups. We did not examine the confidence–accuracy relationship for participants who did not believe that they had consumed alcohol because of sample size limitations.

We further examined the confidence–accuracy relationship using other approaches. We computed the calibration statistic (C) to index the extent to which each beverage group differed from perfect calibration, with the index value ranging from 0, indicating perfect calibration, to 1, indicating poor calibration. We also calculated the overconfidence/underconfidence measure (O/U) for each beverage group, which indexes the overall tendency of participants to respond with confidence that is greater or less than is warranted by their accuracy level. The O/U measure ranges from −1, indicating extreme underconfidence, to +1, indicating extreme overconfidence. Finally, we also calculated the Normalized Resolution Index (NRI), which ranges from 0, indicating no discrimination between correct and incorrect identification decisions, to 1, indicating perfect discrimination. We collapsed across alcohol expectancy in these analyses because expectancy was not significant in any previous analyses.

Table 3 shows the C, O/U, and NRI statistics for the tonic water and the alcohol groups. As can be seen, the two beverage groups performed similarly on the measures. The tendency towards greater overconfidence by the tonic water compared with the alcohol group is reflected in the O/U statistics; however, inspection of the 95% CIs indicates that this difference was not statistically significant.

**Table 3:** Calibration statistics by beverage group, choosers and nonchoosers combined

	Consumed tonic water			Consumed alcohol		
	Value	Jackknife *SE*	95% CI	Value	Jackknife *SE*	95% CI
O/U	−0.32	0.06	−0.43 to −0.20	−0.40	0.06	−0.52 to −0.28
C	0.07	0.15	−0.22 to 0.36	0.04	0.12	−0.17 to 0.27
NRI	0.03	0.03	−0.03 to 0.09	0.04	0.05	−0.06 to 0.14

## Discussion

We tested whether alcohol intoxication during encoding impairs line‐up identification accuracy and confidence–accuracy calibration. We found that identification accuracy did not vary depending on whether participants had consumed alcohol, in line with past research (Hagsand et al., 2013; Harvey et al., 2013; Kneller & Harvey, 2016; Yuille & Tollestrup, 1990). We also found that women were more confident in their identification decision if they made an accurate as opposed to inaccurate identification. Further, women who had consumed tonic water rather than alcohol were more confident about the accuracy of their identification decision overall, regardless of accuracy. Calibration analyses suggested that confidence is predictive of accuracy, regardless of whether women had consumed alcohol or tonic. We discuss these findings in turn.

Our finding that alcohol did not affect the accuracy of line‐up identification is among a growing list of studies finding no effect of alcohol on participant witness' ability to make accurate identifications from a line‐up (e.g., Hagsand et al., 2013; Harvey et al., 2013; Kneller & Harvey, 2016; Yuille & Tollestrup, 1990). One reason why alcohol may not have affected identification accuracy in this study and the others is because the doses employed were not high enough to cause memory impairment. Participants in the Dysart et al. (2002) field study were more intoxicated, with a mean blood alcohol level of 0.09% in the high alcohol condition (range: 0.04–0.20%), which may account for why they found an alcohol‐related decrease in showup identification accuracy. Another reason why we may not have found alcohol effects is that intoxicated and sober participants focused their attention on the perpetrator rather than on more peripheral aspects of the scenario. The alcohol myopia theory framework predicts that alcohol restricts attention to the most salient aspects of a scenario. In this case, any negative impact that alcohol may have had on participants' ability to attend and encode may have been offset by increased attention to and rehearsal of the rape perpetrator. We have found support for this idea in our previous work, which showed that sober and intoxicated women were better able to remember information about the perpetrator compared with more peripheral aspects of a sexual assault scenario (e.g., bystanders; Flowe, Takarangi, Humphries, & Wright, 2016). As others have noted, however, it is difficult to determine which aspects of a scene are considered to be central and peripheral, and the amount of attention paid to central and peripheral items might depend on the complexity of the particular stimulus materials employed (e.g., Harvey et al., 2013).

Further, the hypervigilance hypothesis predicts alcohol and alcohol expectancy leads women to pay greater attention in potentially risky scenarios. We did not find support for this, however, as sober and intoxicated women did not differ in their identification accuracy, regardless of whether they expected alcohol. It is possible that we did not find a hypervigilance enhancement in identification accuracy because the perpetrator was presented before participants deemed the scenario to be potentially risky. Even if participants who expected alcohol paid increased attention when the sexual activity occurred, this would have occurred after the perpetrator's appearance was encoded. Needless to say, further research is required to clarify what conditions lead to hypervigilance.

In other alcohol research investigating line‐up identification confidence, participants who had consumed alcohol as opposed to no alcohol have been found to report, on average, lower confidence (Harvey et al., 2013) and lower confidence for incorrect identifications (Yuille & Tollestrup, 1990), whereas other studies have reported null findings (Hagsand et al., 2013; Kneller & Harvey, 2016). In our study, we found that women reported lower confidence for inaccurate compared with accurate identifications, regardless of whether they were alcohol‐intoxicated during encoding. Further, participants who were sober compared with intoxicated reported higher levels confidence overall, regardless of identification accuracy. These results suggest that women who had consumed alcohol were, generally speaking, underconfident, as they were just as accurate at identifying the perpetrator as those who had consumed tonic water.

Calibration analyses were undertaken to shed light on the effects of alcohol on metacognition. The optimality hypothesis (Bothwell et al., 1987; Deffenbacher, 1980) predicts better confidence–accuracy calibration for sober compared with intoxicated participants. Our findings, however, were not in line with the optimality hypothesis, because the confidence–accuracy relationship did not vary across beverage conditions. We did, however, find a trend for those who expected alcohol and consumed it to be slightly underconfident at the midrange of the confidence scale, while all the other groups were overconfident. While it is important to remember that this difference was not statistically significant, Yuille and Tollestrup (1990) also reported a stronger confidence–accuracy relationship if participants had consumed alcohol as opposed to no alcohol. The ‘theory‐based’ confidence judgment model proposes that under some circumstances, overconfidence will be reduced because participants take into account factors that influence memory when making their confidence judgments. Given our null findings, we are not able to rule out this hypothesis (Palmer, Brewer et al., 2013). Given Yuille and Tollestrup's (1990) findings, however, it seems that the theory‐based model might better account for the findings to date on the confidence–accuracy relationship in intoxicated participants than the optimality hypothesis.

We also found evidence that the physiological effects of alcohol were driving women's confidence ratings. Namely, we varied alcohol expectancy and did not find evidence that identification accuracy or confidence was affected by the mere belief that one had consumed alcohol. These null results, however, may have arisen from the way in which we manipulated alcohol expectancy. Every woman in our study consumed their beverage from a vodka‐rimmed cup that also contained vodka‐soaked limes. This was performed to (i) increase the believability of the alcohol expectancy manipulation for those who were consuming tonic alone, but who were told that they had been given alcohol, and (ii) hold alcohol sensory cues (i.e., smell) constant across all experimental groups. However, the smell of alcohol probably led women in our tonic water group to overestimate the likelihood that they had consumed alcohol. We suggest that future research investigating expectancy effects include two types of tonic water control groups: One group should be told that they are consuming alcohol, but they should be given tonic water alone, without any alcohol sensory cues. The other group should be told that they are consuming alcohol, but they should be given tonic water along with alcohol sensory cues. To the extent that the mere belief that one has consumed alcohol decreases confidence, people in the control group who are exposed to alcohol sensory cues should report relatively low confidence compared with the other control group.

Our study has limitations. First, owing to the complexity and large amount of resources necessary for carrying out this type of research, our sample size—although large relative to most other studies conducted to date on this topic (Table 1)—would ideally have been larger. We hope that other laboratories will replicate our studies and, perhaps, that a meta‐analysis across studies will be conducted. Second, for ethical reasons, the scenario was presented in a written format, accompanied by a photograph of the assailant, rather than in say, a video format. On the one hand, one could argue that the psychological realism of our approach was quite high (Mook, 1983). On the other hand, like with most other psychology research, we have no way of knowing whether the memory processes elicited by our procedures are externally valid. We know of no research that can settle the issue. Third, victims in actual cases may be considerably more alcohol‐intoxicated than our participants were and have ingested alcohol as well as other drugs. A recent study by Hagemann et al. (2013) measured serum blood alcohol concentrations for real world rape victims who consulted to a Sexual Assault Centre within 12 hours after the assault. They performed back‐calculations to estimate BAC at the time of the assault and found a median BAC of 0.20% (range: 0.04–0.39%). Additionally, 5% had ethanol along with another drug in their system (e.g., benzodiazapines). We would encourage other researchers to conduct research in public houses (e.g., following Dysart et al., 2002) because it permits researchers to capture memory performance under relatively high levels of intoxication than might be feasible or ethical in the laboratory. Fourth, the accuracy levels we observed may be specific to the particular materials we employed. It is reasonable to expect that identification accuracy varies in relation to encoding conditions. In the present study, the hypothetical assailant was presented in a photograph during encoding, and participants viewed the photograph for about 8–10 seconds. Despite the fact that our exposure time was relatively short, identification accuracy was relatively high. Future studies should systematically control exposure duration length, perhaps tracking eye movements to ensure that intoxicated and sober participants attend to the face for the same length of time. Studies that employ longer exposure times are needed to estimate real world accuracy levels. Fifth, we did not systematically vary whether participants were tested in a sober versus intoxicated state because of resource limitations. Even though our participants were reportedly sober when they completed the recognition measures, they completed the recognition measures online, and hence, we could not breathalyze them to verify that they were in fact sober. Thus, we cannot rule out the possibility that state dependency affected identification accuracy or confidence. Nevertheless, state dependency effects tend to be small and idiosyncratic (Duka, Weissenborn, & Dienes, 2001; Weissenborn & Duka, 2000) and seem to affect recall rather than recognition (Eich, 1980). Sixth, we did not find that women were less accurate if they were tested after a 7‐day compared with a 1‐day delay. We suggest that future studies employ a longer delay between event encoding and the line‐up identification test. Finally, we tested participants online. Previous line‐up research finds a similar pattern of results with respect to discrimination accuracy for participants who are tested online versus in the laboratory (e.g., Flowe, Takarangi et al., 2016; Gronlund et al., 2009; Mickes et al., 2012). It is entirely conceivable, however, that decision bias (i.e., the willingness to identify someone as a suspect from a line‐up, which is a separate issue from discrimination accuracy) might differ depending on testing context. Further, unlike a laboratory or police environment, testing conditions (e.g., noise and other distractions, time of day) cannot be precisely controlled when participants are tested online, and this may have affected accuracy and decision bias.

Bearing in mind these limitations, how might our results inform investigative practice? Alcohol would seem to be an especially pertinent issue to consider when assessing whether a witness' identification is likely to be accurate. The relevance of the issue is reflected in a survey of psychology experts, which found that 90% of respondents agreed that alcohol impairs memory performance (Kassin, Tubb, Hosch, & Memon, 2001). Further, 61% of respondents on this survey indicated that they would testify about it, 79% indicated there was a sufficient research basis, and 95% indicated that the phenomenon was common sense (Kassin et al., 2001). Mock jurors have also been shown to perceive intoxicated victims and bystanders as being cognitively impaired and less able to make accurate identifications when evaluating summaries of sexual battery and aggravated sexual assault cases (Evans & Schreiber Compo, 2010). However, the results of the current study add to a growing body of research (Hagsand et al., 2013; Harvey et al., 2013; Kneller & Harvey, 2016; Yuille & Tollestrup, 1990) which finds that people who are alcohol‐intoxicated (BAC = 0.04–0.11%) compared with sober during encoding can be just as accurate on a line‐up test. To put these BAC levels into perspective, a BAC of 0.08% is equivalent to consuming about four regular sized alcoholic drinks (beer, wine, or shot) in an hour. Our work, along with others, suggests that investigators should not automatically exclude line‐up identification evidence or discount a confidence statement because it was obtained from a witness who was intoxicated with alcohol during the crime. Having said this, the witness' intoxication state at the time of identification test is an important factor to consider. Like the studies before us, we found no differences between sober and intoxicated participants when they were tested between 24 hours to a week after the crime. An outstanding issue in need of empirical investigation is whether witnesses, if they are intoxicated during the line‐up test, can follow line‐up instructions (such as warnings that the perpetrator might not present in the line‐up, e.g., Clark, 2005) or whether they are more subject to potentially biasing influences during the line‐up (e.g., Clark, Marshall, & Rosenthal, 2009). Further, previous work suggests that witnesses should not be tested with a showup when they are intoxicated, because intoxicated compared with sober witnesses are more likely on average to false alarm on a showup test (Dysart et al., 2002). Finally, our results demonstrate that, for sober and intoxicated women alike, identifications made with low compared with high confidence are more likely to be incorrect. This result is particularly important for criminal investigations. In post‐conviction DNA cases, witnesses expressed low levels of confidence in their identification at the time that they took the line‐up test (Garrett, 2011). Yet, over time, witnesses became increasingly confident that they had identified the perpetrator. This finding further underscores the necessity of taking confidence at the time that the identification is made to ensure that confidence is a valid indicator of memory strength (e.g., Bradfield, Wells, & Olson, 2002).

In sum, we found that women who had consumed alcohol (BAC = 0.08%) compared with tonic before encoding were just as able to identify the rape assailant from a line‐up from 1 to 7 days later. Women who had consumed alcohol as opposed to tonic water tended to identify the assailant with lower confidence, however. Even still, confidence and accuracy were related in a similar manner for both the alcohol and tonic water groups.
